# Transformation and functional verification of *Cry5Aa* in cotton

**DOI:** 10.1038/s41598-021-82495-8

**Published:** 2021-02-02

**Authors:** Shihao Zhao, Feng Wang, Qiuping Zhang, Jiayi Zou, Zhangshu Xie, Kan Li, Jingyi Li, Bo Li, Wen He, Jinxiang Chen, Yunxin He, Zhonghua Zhou

**Affiliations:** 1 Institute of Cotton Science, Hunan Agricultural University, Changsha, China; 2 College of Agronomy, Hunan Agricultural University, Changsha, China; 3 Hunan Institute of Cotton Science, Changde, China

**Keywords:** Plant physiology, Molecular biology, Plant sciences

## Abstract

Most of the cotton bollworm-resistant genes applied in cotton are more than 20 years and they all belong to *Cry1Ab/c* family, but the insect-resistant effects of *Cry5Aa* on cotton were rarely reported. The possible risk of resistance is increasing. The study synthesized a novel bollworm-resistant gene *Cry5Aa* artificially based on preferences of cotton codon. The new gene was transferred to cotton through the method of pollen tube pathway. The transgenic strains were identified by kanamycin test in field and laboratory PCR analysis. Meanwhile, an insect resistance test was conducted by artificial bollworm feeding with transgenic leaves and GK19 was used as a control in this study. Results showed that rate of positive transgenic strains with kanamycin resistance in the first generation (T1), the second generation (T2) and the third generation (T3) respectively were 7.76%, 73.1% and 95.5%. However, PCR analysis showed that the positive strain rate in T1, T2 and T3 were 2.35%, 55.8% and 94.5%, respectively. The resistant assay of cotton bollworm showed that the mortality rate of the second, third and fourth instar larva feed by the transgenic cotton leaves, were 85.42%, 73.35% and 62.79%, respectively. There was a significant difference between transgenic plant of *Cry5Aa* and GK19 in insect resistance. Finally, we also conducted the further analysis of gene expression patterns, gene flow and the effect on non-target pest in the study. The results showed that *Cry5Aa* gene had less environmental impact, and *Cry5Aa* has been transferred successfully and expressed stably in cotton. Therefore, the novel bollworm resistance gene can partially replace the current insect-resistance gene of Lepidoptera insects.

## Introduction

*Bacillus thuringiensis* (*Bt*) transgenic insect-resistant cotton has been widely planted in China, and this has resulted in the efficient control of cotton bollworm populations with reduced use of insecticides^1,2^. However, evolution of resistance by bollworm(s) threatens the continued planting of single gene *Bt* cotton^3,4^. Co-expressing of different types of insect-resistant *Bt* genes in cotton could alleviate the resistance of bollworm to transgenic insect-resistant cotton and extend the lifespan of transgenic cotton.

*Cry5Aa* is a member of *Bt* family, is a crystal protein produced by the *Bacillus* serotype^5^, highly toxic to root-knot nematodes and some lepidopteran pests. Lots of studies have shown that *Cry5Aa* shares a three dimensional structure with *Cry1Aa,* but it has long insertions in a2 of domain I. The particular extension may involve in pore formation and specific determination, and some of the rings in domains II and III are exposed, which makes it important for the design of high-efficiency bio-nematicides^5^. Transferred *Cry5Aa* into maize and potato could or has improved plants resistance to nematodes. The binding site of *Cry5Aa* to the nematode glycolipid receptor oligosaccharide fragment or fragments is between crystal protein domain I and domain II, because the interaction between *Cry5Aa* and oligosaccharide fragments is mainly conceived, followed by hydrogen bond energy, and static electricity. The action energy is zero, and a stable complex can be formed between them, which are more favorable for understanding the toxicological mechanism of the crystal protein *Cry5Aa* of B. *thuringiensis* and improving the nematicidal activity of *Cry5Aa*^6^.

In view of the fact that *Bt* gene has a good toxicity effect on lepidopteran pest, and the fact that *Cry5Aa* has not been studied in cotton, the purpose of our study was to transform the recombinant plasmid which contain the novel *Bt* insect-resistant gene, *Cry5Aa*, into the cotton by using the pollen tube pathway method.

We screened and identified the progeny using molecular biology method to verify the transfer of *Cry5Aa Bt* gene. We also conducted the further analyze of its expression patterns in cotton and analyze gene flow in environment and the possible effect on non-target pest. This study provided a new exploration for cotton transgenic insect resistance and lays the foundation for the creation of new insect-resistant germplasm resources.

## Materials and methods

### Materials

**Figure 1:**
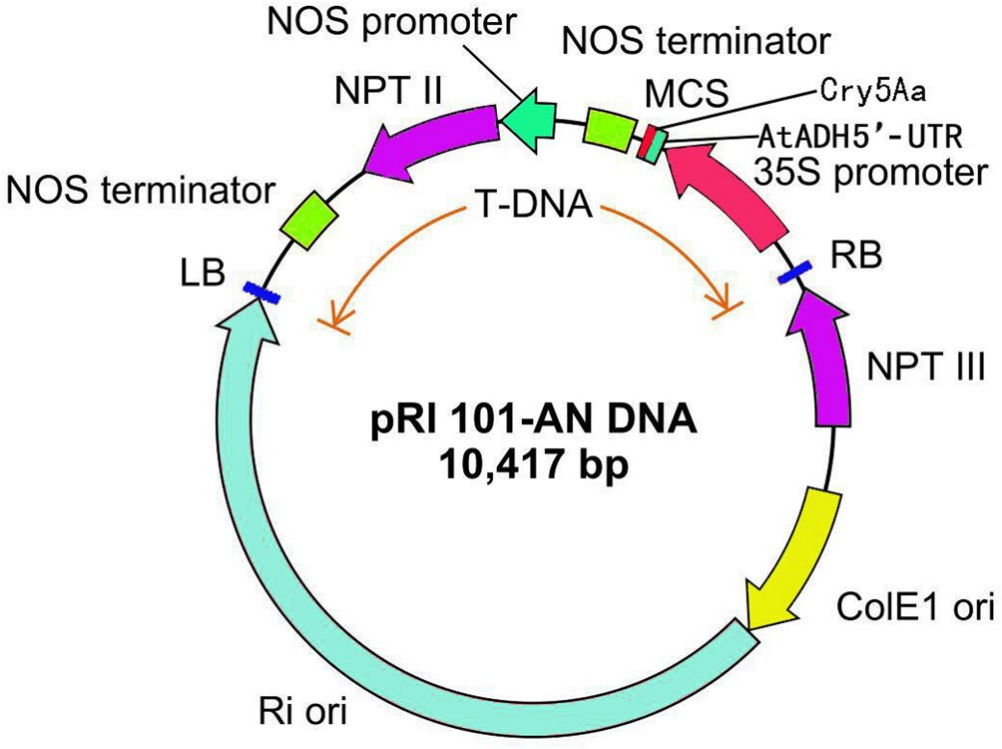
Plasmid construction of *Cry5Aa.*

*Cry5Aa* was synthetized cording the sequence of L07026.1 published in Genebank by Sangon Biotech (Shanghai) Co., Ltd. Jin1, a non-transgenic cotton line was provided by Institution of Cotton Science, Hunan Agricultural University. PRI101-An-*Cry5Aa*, the plasmid contains *Cry5Aa Bt* gene was constructed by Institution of Cotton Science. The maps of plasmid are showed in Fig. 1. The gene was fused together with HindIII/SphI.

### Pollen tube pathway

The methods of pollen tube pathway were defined as described by Zhao et al.^7^. The plasmid DNA was extracted, purified and diluted to the concentration of 100 μg/μL. On the next day of flowering, the flowers of the first and second fruit nodes of each fruit branch were selected as the objects of transgenic operation. The petals were stripped off and the plasmid was pushed into fertilized ovary with a 50 μL medical micro injector and each ovary was injected with 5 μL of diluted plasmid.

### Kanamycin test

After harvesting the seeds of T_0_ generation, we sowed them in Sanya, Hainan Province, China in November 2011. We applied floating nursing for seeding and transplanted them to the field when they reached the three-leaves stage. We employed kanamycin to identify transgenic Bt cotton plant in the field experiment because the selection marker of NPT II involved in the plasmid produces kanamycin resistance in the leaves of transgenic cotton plants. The expanded leaves at seedling stage were scribbled with a writing-brush soaked in 1.6 μg/μL of kanamycin solution. Result was revealed three days later because all of leaves of non-transgenic plants showed apparent yellow plaque whereas the leaves of *Bt* transgenic plants remained green without any symptom.

### Genomic DNA extraction and PCR analysis of the transgenic plants

The total DNA of cotton leaves were extracted by the method of CTAB. Based on the *Cry5Aa* gene, the primers were designed by Primer 5.0, just as follow: 5′-GCCATCCAGGTTGCTATCT-3′, 5′-GCTGACGAAAGAATGCTGA-3′. The target gene was 697 bp long. The total volume of the PCR reaction system was 20 μL. The concentration of template DNA was 10 μg/μL. The content of template DNA in reaction system was 20 μg. In this reaction, we added 2 μL of 10× taq buffer and a touchdown protocol was employed with the annealing temperature about 95 °C, following by 30 cycles at 58 °C and a 45 s of extension time at 72 °C for all cycles. After that, samples were conserved at 4 °C.

### The analysis of *Bt* toxic protein in different organs and tissues

According to specific peptide sequence CNPNQPCKDDLDRV, the specific antibody was prepared by Nanjing Kingsri Bioengineering Co, Ltd. The total protein was extracted from cotton leaves at different stage by TCA-acetone precipitation. A standard curve was produced with 0.25–4.0 × 10^–6^ μg/μL of *Cry5Aa* crystal protein and PBST buffer was used as a control. OD value of *Bt* toxic protein extracted from different organs and tissues of transgenic cotton lines were detected by ELISA assay. The contents of toxic proteins in different cotton organs and tissues were obtained according to the standard curve.

### The test of insect-resistance

The cotton bollworm resistant assay of four *Cry5Aa* transgenic line including JX0010, JX0020, JX011 and JX012 was conducted. Jin 1 and GK19 were used as negative and positive control, respectively. Jin1 is the receptor and GK19 is a *Cry1Ac Bt* transgenic cotton variety widely planted in China. At the peak of the second, third and fourth generation of bollworm larvae, 20 cotton plants were randomly selected from each transgenic line and the control, and the leaves on the top of each plant were collected, marked, and brought back to the room in an ice box. The collected cotton leaves were put into a plastic box with a diameter of 6 cm and a height of 4 cm. One leaf was placed in each box. Five 1-day-old cotton bollworm larvae were injected into the box. After catching the worm, tighten the lid of the box. Culture under the condition of 25 ± 1 °C and 50–80% RH, and the survival rate was detected 5 days later. During examination, if the worm does not respond to the light touch with the tip of a soft brush, it will be recorded as death. The mortality rate was calculated.

### Gene flow monitoring of *Cry5Aa*

The monitoring test of gene flow of *Cry5Aa* transgenic cotton was conducted at Heba Town, Datonghu District, Yiyang City, Hunan Province, China. Experiment area was about 12,000 m^2^. JX0010, one of *Cry5Aa* transgenic lines was planted in the centre with an area of 25 m^2^, and the other test area was planted with non-transgenic variety named Xiangmian 10. Referring to some related governmental regulations on environmental safety assessment and gene flow monitoring of transgenic plants in China. We marked four monitoring area A, B, C, D that the distance between it and the area of transgenic cotton were 5 m, 15 m, 30 m and 60 m, respectively. A_1_–A_4_, B_1_–B_4_, C_1_–C_4_ and D_1_–D_4_ represent separately four directions of the experimental field. Total ten cotton plants were randomly picked at each point and marked their order such as A_1_, A_2_, A_3_ or A_4_. After the seed harvested from picked plants cotton being air-dried, they were individually ginned and conserved for further testing.

These seeds were planted in the greenhouse of Hunan Agricultural University in Changsha, Hunan. After that, the genomic DNA of leaves at seedling stage were extracted and identified by PCR with *Cry5Aa* Bt specific primers and rate of positive *Cry5Aa* seeds to all seeds were calculated. Finally, gene flow of *Cry5Aa* was determined according to spreading distance of its pollens.

### Effect of *Cry5Aa* on non-target pests

The experiment was conducted in 2010 at the experimental station of Hunan Agricultural University, Changsha, Hunan Province. Four non-target pests except the target pest bollworm including cotton aphid, miridae, two-spotted spider mite and cotton thrip were investigated every 20 days from May 20 to September 20 in field planted with *Cry5Aa* transgenic line JX0010 and non-transgenic control Xiangmian10. The *Cry5Aa* transgenic line and the control were both cultivated and managed in accordance with cotton cultivation technical standard of Hunan Province.

## Results

### Transformation of *Cry5Aa* to cotton

The plasmid containing the *Cry5Aa* gene was injected into young cotton bolls by pollen tube pathway and 0.6 μg/μL of gibberellins solution was sprayed onto bract for boll protection. The average setting rate of bolls reached 73.3%. A total of 2529 seeds were obtained for further identification (Fig. 1).

### Kanamycin test and PCR analysis of transgenic lines

**Figure 2:**
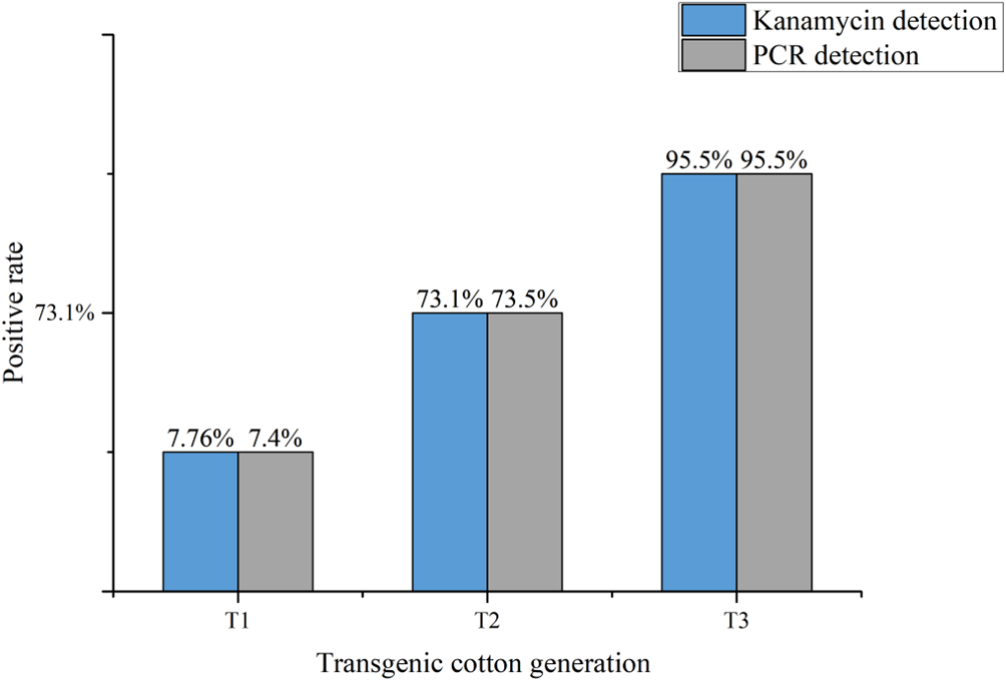
Kanamycin test and PCR analysis of *Cry5Aa* transgenic line.

After the seeds of To generation were harvested in 2010, they were planted at the research station of Hunan Agricultural University in Sanya, Hainan during the winter of that year. Total 1829 seeds of T_1_ generation germinated normally while the other seeds failed to survive. The expanded leaves were sprayed with 1.6 μg/μL of kanamycin solution at seedling stage for preliminary identification. Total 142 plants without yellow plaque indicated that the positive rate of *Cry5Aa* transgenic line by kanamycin test was 7.76%. Meanwhile, PCR analysis was performed after genomic DNA extraction from transgenic plants. Specific band were identified in 140 plants showed that the positive rate was 7.4% by PCR analysis (Fig. 2). There was no significant difference between kanamycin test and PCR analysis proved that kanamycin test could be a simple, cheap and efficient technique for gene identification at least for cotton.

**Figure 3:**
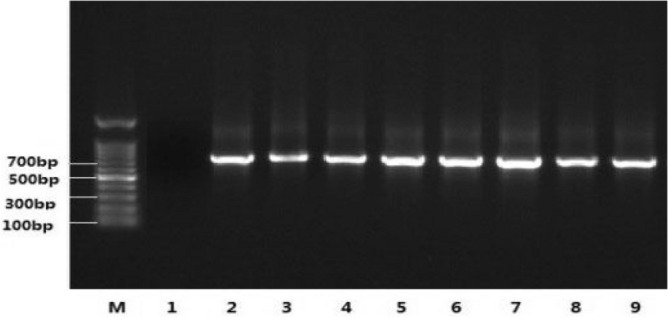
PCR analysis of *Cry5Aa* transgenic line. M: DNA Mark; 1: Non-transformed Control; 9: positive control; 2–8: *Cry5Aa* transgenic lines.

The seeds of T_2_ generation were harvested from positive T_1_ transgenic plants. A total of 845 T_2_ plants were obtained at cotton base of Hunan Agricultural University in Changsha, Hunan in the same year. There were 618 positive plants without yellow spots after spraying with kanamycin solution. While the positive rate of transgenic line was 73.1% by kanamycin test and the positive ratio detected by PCR was 73.45% (Fig. 3).

The seeds of T_3_ generation were obtained from positive T_2_ plants and total 360 T_3_ plants were planted. There were 360 positive plants without yellow plaque after spraying with kanamycin solution. The rate of positive strain was 95.5% and the positive rate detected by PCR was 95.5%.

### The expression character of *Cry5Aa*

Since the *Cry5Aa* is a novel *Bt* gene, there was no any ideal detection method could be applied. A specific antibody to *Cry5Aa* toxin protein was synthesized for ELISA analysis. The expression level of toxic protein in various tissues from T3 generation of transgenic line JX0010 at different stages were determined by the method of ELISA.

A standard curve was produced with purified *Cry5Aa* crystal protein and it was diluted to the concentration of 0.25, 0.5, 1.0, 2.0 and 4.0 × 10^–6^ μg/μL, respectively by PBST buffer. PBST buffer was used as a blank control. It can be seen that the regression equation of the standard curve for *Cry5Aa* toxin protein was Y = 0.4093x + 0.1496. The correlation co-efficient R2 was 0.998 which indicates that OD value was significantly correlated with the content of *Cry5Aa* standard protein.

**Figure 4:**
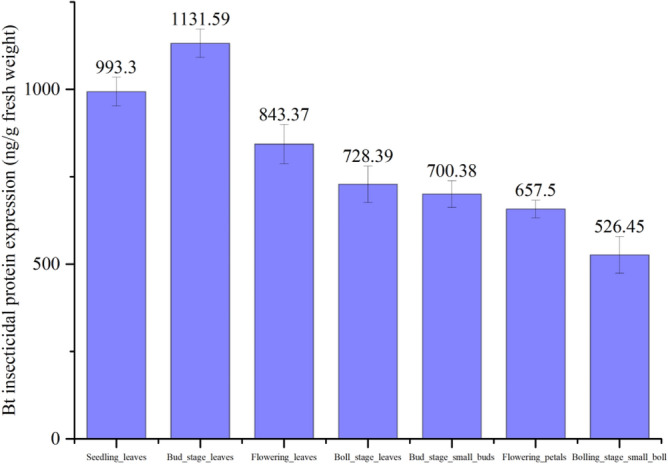
Gene flow monitoring of *Cry5Aa*.

Through the ELISA detection of *Cry5Aa* toxin protein, the protein expression level of various stages and different tissues were shown in Fig. 4. *Cry5Aa* protein content of the leaves at bud stage was 1131.59 × 10^–6^ μg/μL and it was the highest expression level. However, toxin protein content of small bolls at boll stage was with the lowest level of 526.45 × 10^–6^ μg/μL. The results showed that *Cry5Aa* toxin protein was with the highest expression level at the bud stage among all developmental stages which explained its time distribution characteristics. In terms of the spatial distribution character of the novel *Bt* protein, it displayed the highest expression level in leaves among all tissues.

### Determination of bollworm-resistance of *Cry5Aa* transgenic lines

**Figure 5:**
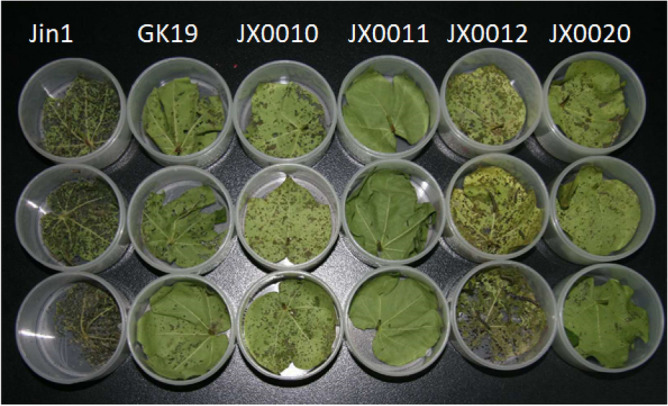
The bollworm resistances of transgenic line.

The results showed that the adjusted death rate of fed cotton bollworm of four *Cry5Aa* transgenic lines including JX0010, JX011, JX012 and JX0020 was 82.00%, 100%, 69.50% and 76.50%, respectively (Table 1, Fig. 5). However, that of the control GK19 was only 68%. The adjusted death rate of fed cotton bollworm of transgenic line JX0010 and JX011 increased significantly at 0.05 level compared with the control and the damage degree index of the leaves was 1. There was no significant difference between the other two transgenic lines JX012, JX0020 and the control with the same damage degree index of 2.

**Table 1:** The bollworm larva resistance of *Cry5Aa* transgenic lines.

Bt transgenic lines	Corrective mortality (%)	± (%)	Leaf damage index
JX0010	82.00 ± 3.00ba	14.00	1
JX011	100.00 ± 0.00bB	32.00	1
JX012	69.50 ± 1.50aA	1.50	2
JX0020	76.50 ± 4.50aA	8.50	2
GK19	68.00 ± 2.00aA	0.00	2

Lowercase or uppercase letters represent significant difference at 0.05 or at 0.01 levels, respectively.

### Gene flow of *Cry5Aa* transgenic line

The result showed that the maximum pollen spreading distant of *Cry5Aa* transgenic line JX0010 was 30 m, which indicated gene flow of the novel *Bt* gene is limited. There was no difference among gene flow along four different directions within 15 m (Table 2). While the difference was detected in monitoring spots among different directions with 30 m distance to transgenic area, which could be explained by the possible influence of the wind direction. The pollen of transgenic cotton can flow to the north farther away in the test site because of more south winds in summer.

**Table 2:** Gene flow of *Cry5Aa.*

Spot	Pollen spreading distance (m)	Positive Cry5Aa seeds (%)				Average value
		1	2	3	4	
A	5	2.53	1.99	2.03	2.38	2.23
B	15	1.42	1.03	1.51	1.48	1.36
C	30	0.94	0.48	0	0	0.36
D	60	0	0	0	0	0

### Effect of *Cry5Aa* on non-targeted pests

The experimental results illustrated the populations of all four tested non-target pests including cotton aphid, miridae, two-spotted spider mite and cotton changed among different growth and development stages. However, there was no significant difference between *Cry5Aa* transgenic line JX0010 and the non-transgenic control Xiangmian10, which maybe indicated that the novel *Bt* gene had less influence on these non-target pests.

### Cotton aphid

**Figure 6:**
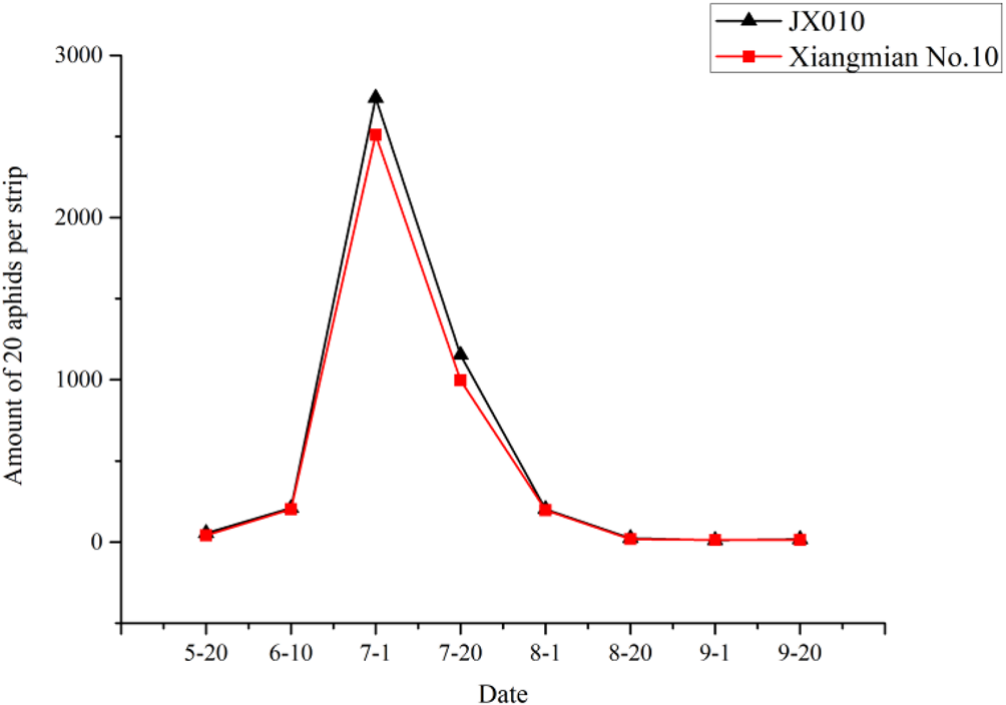
Population dynamics of cotton aphid.

During the entire developmental stage of cotton, the number of cotton aphids changed significantly. The damage extent of cotton plants affected by cotton aphid in transgenic cotton was even more serious than the control, but the difference between them was not significant (Fig. 6).

### Miridae

**Figure 7:**
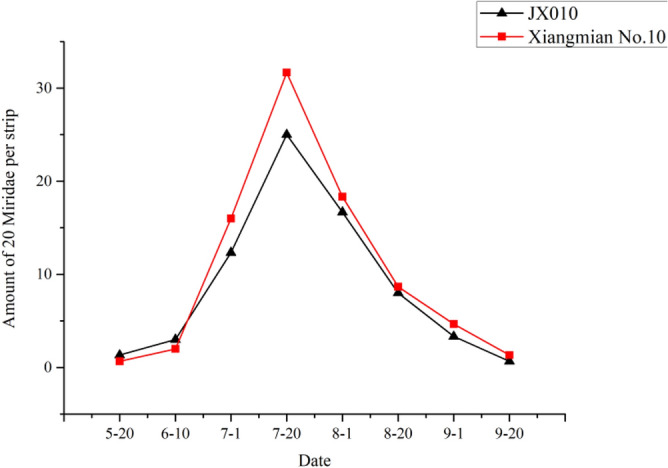
Population dynamics of miridaes.

The population of miridae in transgenic species and non-transgenic cotton are shown in Fig. 7. Miridae appeared in late June and reached the maximum population in late July. While the increasing trend of the transgenic line and the control was similar. *Cry5Aa* transgenic line exhibited lighter damage extent compared with the control, but there was no significant difference between them.

### Two-spotted spider mite

**Figure 8:**
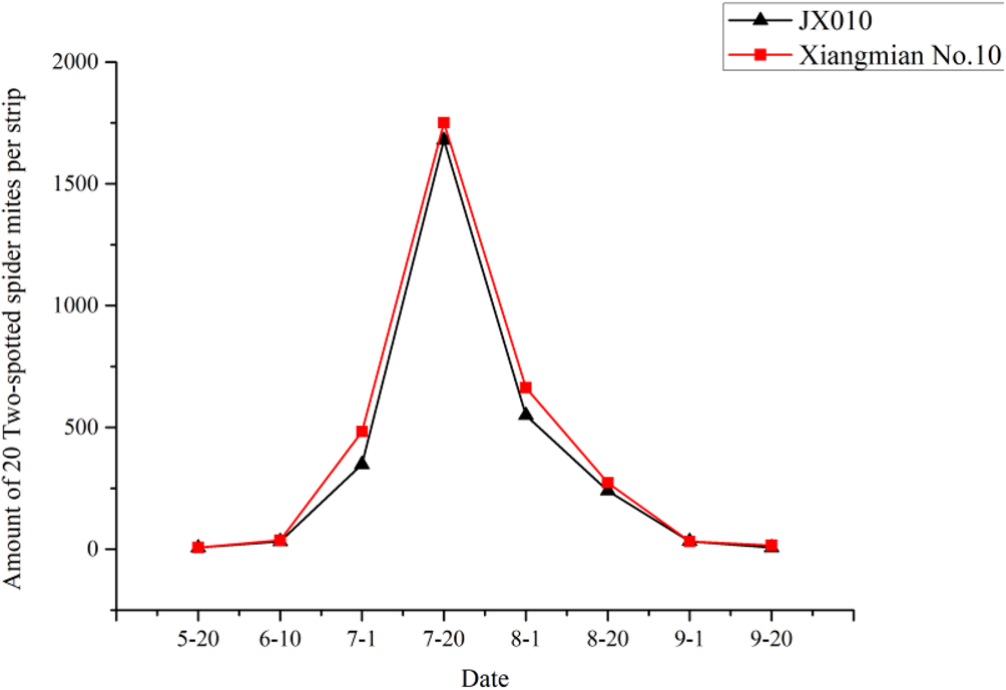
Population dynamics of two-spotted spider mite.

The population of two-spotted spider mite in both transgenic and non-transgenic cotton showed similar changing trend during developmental stages (Fig. 8). The pest occurred in late June, then the population increased gradually and reached to the peak value in late July. Compared with the control, the damage caused by two-spotted spider mite in transgenic lines JX0010 were slighter, but there was no significant difference between them.

### Cotton thrips

**Figure 9:**
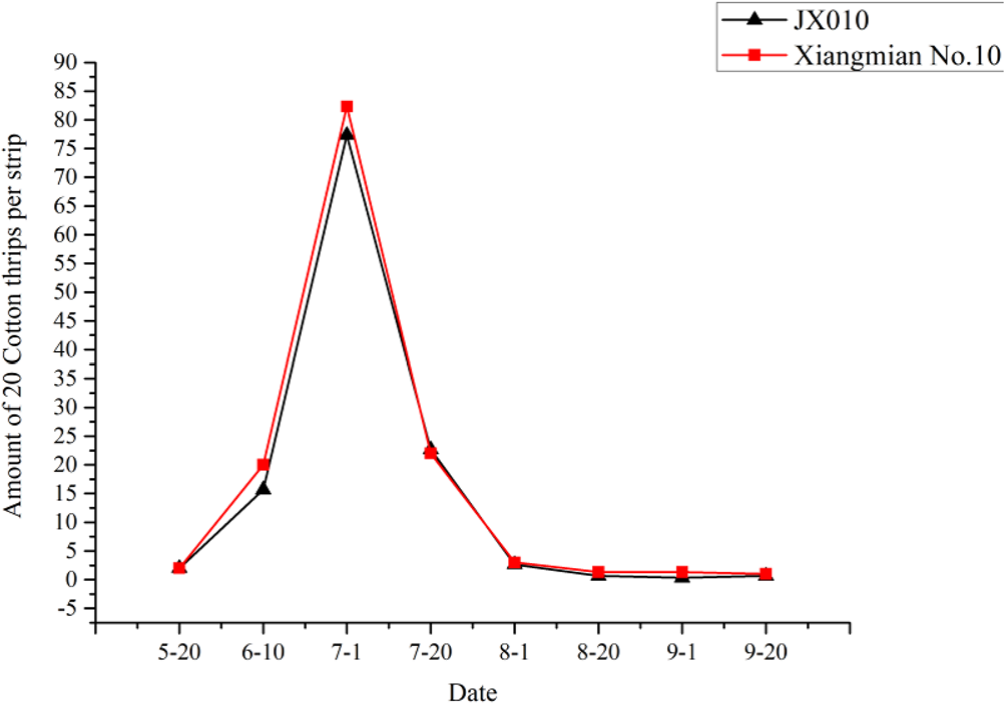
Population dynamics of cotton thrips.

Cotton thrips occurred in late May, its population reached to the peak value in late July, and the population size decreased gradually (Fig. 9). There was almost no difference in the degree of damage between transgenic strain JX0010 and the control.

## Discussion

In 1983, Guangyu Zhou first transferred the foreign DNA into cotton through the pollen tube pathway method to obtain transgenic cotton plants with resistance to blight^8^. *Bt* insect-resistant transgenic cotton were obtained by this method for the first time was reported in 1991^9^.

The pollen tube pathway method has the advantages such as no genotype limitation, no need of tissue culture, easy operation for large scale of plant transformation, etc^10^. The transformation rate was as high as 7.4% in this study proved the reliability of the method. Of course, the pollen tube pathway method is time consuming and requires rich experience of fertilization in cotton. Moreover, the most appropriate time for transformation is 18–48 h after pollination^11^. In addition, the conclusion can be drawn by this study, that the delayed transformation process in the early August combined with gibberellin spraying onto bracts can significantly reduce the shedding of cotton bolls, and effectively increase the transformation rate.

The novel *Bt* gene of *Cry5Aa* hasn’t been reported the introduction to cotton. The specific primers were designed for PCR that used to confirm the transformation of the exogenous *Cry5Aa* in transgenic cotton. Firstly, the transgenic lines were preliminarily identified by kanamycin test in field. Then those positive transgenic plants were confirmed by laboratory PCR analysis. The positive rates identified by the two different methods were approximated proved that kanamycin test could be an effective, convenient and fast methods for transgenic confirmation. Moreover, high concentrations of kanamycin used in this study, which may be necessary for transformation confirmation and can significantly improve the accuracy of the assay.

The results of ELISA assay confirmed that *Cry5Aa* was indeed expressed in various tissues at different developmental stages of transgenic line JX0010. There was a significant difference in the expression levels of various tissues, which is consistent with the previous studies on expression character of other *Bt* genes. This study confirmed that the insecticidal effect of *Cry5Aa* transgenic lines on cotton bollworm and their insect resistances had a tendency to decrease along with the growth and development process of cotton plants^12–14^.

The bollworm resistance test of four *Cry5Aa* transgenic lines showed higher insect resistance compared to the control. Among four *Cry5Aa* transgenic lines including JX0010, JX011, JX012 and JX0020, the two transgenic lines JX0010 and JX011 significantly increased the bollworm resistances and the damage degree index of the leaves was 1, whereas there was no significant difference between the other lines JX012, JX0020 and the control with the same damage degree index of 2.

Controlling the gene flow of GM crops can reduce the negative impact on non-transgenic crops^15^. The mechanism of action of *Bt* on target pests is that companion crystals are produced during the sporulation stage, and the crystals are taken by lepidopteran insects. After eating, it is activated by the hydrolysis of intestinal enzymes, and Howard toxin can bind to the midgut specific receptors to cause the formation of mesenteric pores and cause insects to die^16^.

Studies have shown that due to the incompatibility of hybridization, unoccupied flowering or geographical isolation, the possibility of gene drift or gene introgression of *Bt* gene transgenic cotton to wild cotton or relative plants of land cotton is basically non-existent^8^. It is important to determine the gene flow distance of the novel *Bt* gene by field experiment. The results that the maximum pollen spreading distant of *Cry5Aa* transgenic line was limited, indicates that the *Cry5Aa* has less impact on the environment. During the growth and development of *Cry5Aa* transgenic cotton, it was found that the populations of all four non-target pests such as cotton aphid, miridae, two-spotted spider mite and cotton changed along with various development stages of transgenic cotton plants. However, there was no significant difference between *Cry5Aa* transgenic line JX0010 and the non transgenic control. Previous paper reported that *Cry5Aa* toxin has a certain inhibitory effect on blind mites and double-spotted spider mites^17^. Some other factors such as cotton varieties, management measures, and geography also play vital roles in impact of transgenic lines on non-target pests In the future, prevention of possible harm on non-target insects caused by transgenic *Bt* cotton is as important as control of cotton bollworms.

## Conclusion

In this study, the transformation and functional confirmation of *Cry5Aa* was introduced in detail. *Cry5Aa*, as a novel *Bt* insect-resistant gene, can significantly improve the resistance of transgenic plants to cotton bollworm, and can partially replace the current mainstream lepidopteran resistance gene. At present, the safety of transgenics is the most concerned issue. This study confirms that *Cry5Aa* can be safely applied to production and has controllable environmental impact. Due to the effective control of lepidopteran pests such as cotton bollworm and the economic and ecological benefits of the production, the transgenic *Bt* cotton has been accepted by more and more people, which has made it impossible to maintain the sustainable development of cotton in the world.
